# Liver Medicine

**Published:** 1876-11

**Authors:** 


*Liver Medicine.*—The liver is the best abused organ of the body. It is cursed for doing things of which it is innocent, and drugged for maladies to which it is a perfect stranger. However, liver medicines are in vogue, and often the baffled physician, when cornered for a diagnosis, will say, "Oh! it's the liver—you must take some liver medicine!" Everybody has heard of Simmons' Liver Medicine and Regulator, and the good people have consumed gallons of them. We have, by accident, come across the formula for the original Simmons liver medicine.

Over forty years ago Dr. Simmons, of Georgia, while *en route*, as the story goes, to another State, chanced to stop over night at the house of a sick gentleman. The doctor prescribed his liver remedy as usual, it is supposed, and gave him the formula for preparing it. The gentleman—so it is said—was so pleased with the remedy that he used it during his life, and left it, "highly recommended," to his children—some of them using it to this day. From one of these, indirectly, we obtained the formula, which, in good old Anglo-Saxon, is as follows:

R.—Virginia snake root.......................................... oz.i
Senna leaves............................................ oz.ij

Put these in a quart of boiling water, simmer on the fire awhile, and when cool add:

Alcohol.................................................Oss.

S—A tablespoonful three times a day.

If our readers should go for the "liver" with this formula, after this, do not hold us responsible if they fail for furnishing them with the weapon. It is not ours, but from what we can gather, is the original Dr. Simmons liver medicine. It may, however, be of some value and "worthy of a trial"—especially by those who have all "liver" and nothing else, the source of their trouble. G.

